# First school year tapping predicts children's third-grade literacy skills

**DOI:** 10.1038/s41598-023-29367-5

**Published:** 2023-02-09

**Authors:** Csaba Kertész, Ferenc Honbolygó

**Affiliations:** 1 Institute of Psychology, ELTE Eötvös Loránd University, Budapest, Hungary; 2 Institute of Psychology, Károli Gáspár University of the Reformed Church, Budapest, Hungary; 3 Brain Imaging Centre, Research Centre for Natural Sciences, Budapest, Hungary

**Keywords:** Psychology, Human behaviour

## Abstract

Rhythmic skills have been repeatedly found to relate to children’s early literacy skills. Using rhythmic tasks to predict language and reading performance seems a promising direction as they can be easily administered early as a screening test to identify at-risk children. In the present study, we measured Hungarian children’s (N = 37) general cognitive abilities (working memory, non-verbal reasoning and rapid automatized naming), language and literacy skills (vocabulary, word reading, phonological awareness and spelling) and finger tapping performance in a longitudinal design in the first and third grades. We applied metronome stimuli in three tempi (80, 120, 150 bpm) using a synchronization-continuation paradigm and also measured participants’ spontaneous motor tempo. While children’s synchronization asynchrony was lower in third than in the first grade, with the exception of the slow-tempo trials, tapping consistency and continuation tapping success showed no development in this period. First-year tapping consistency in the slow-tempo tasks was associated with third-year reading and spelling outcomes. Our results show that the relation between tapping performance and literacy skills persists throughout the third school year, making the sensorimotor synchronization task a potentially effective instrument for predicting literacy outcomes, and a useful tool for early screening of reading difficulties.

## Introduction

Ample evidence supports the association between early rhythmic skills, language and literacy. Individual differences in children’s non-linguistic rhythmic skills have been shown to relate to language and literacy outcomes in several studies with typically^1–7^ and atypically developing children, including individuals diagnosed with developmental dyslexia (DD)^8–15^ and developmental language disorder (DLD)^16–19^. While phonological awareness^20–22^, short-term verbal memory^23–25^ and rapid automatized naming (RAN)^22,26^ are considered reliable predictors of reading outcomes, many studies have found rhythmic skills to account for unique variance of literacy skills^5,7,27^.

Rhythmic processing seems to play an important role in both musical and language domains. Although they rely on a different set of elements (pitch, rhythm, timbre, stress) and differ in the level of periodicity, music and language both have similar acoustic features and create hierarchical structures that allow the brain to make top-down predictions facilitating perception^28^. A link between the two domains seems to be neural entrainment of brain oscillations to external stimuli and between sensory and motor areas. According to the Dynamic Attending Theory (DAT)^29,30^, entrainment to temporal regularities of auditory input helps efficient processing by directing attention to relevant information. Building on this notion, more theories have emerged on how the brain can capitalize on rhythmic cues when processing speech and music. The sound envelope processing and synchronization and entrainment to pulse hypothesis (SEP)^31^ emphasizes that music processing by training sensorimotor coupling enhances speech perception. According to the precise auditory timing hypothesis (PATH)^32^ precise neural timing plays a key role in both phonological skills and the ability to entrain to the beat of music. Patel and Ozernov-Palchik^28^ came to a similar conclusion stating that the ability to extract temporal regularities from an auditory stream and to make predictions based on these is the mechanism connecting beat perception and phonological awareness. Supporting their claim, both dyslexic children and adults have been shown to perform significantly poorer in contrast to individuals without DD when making timing predictions based on predictable but not on unpredictable stimuli^33^. Establishing a framework for findings with atypical developing children, the Temporal Sampling Framework (TSF)^34^ claims that imprecise entrainment of brain oscillations to auditory stimuli as a domain-general mechanism results in impaired entrainment to speech envelope^13,35^, resulting in imprecise phonological representations and consequently atypical reading, while the same impairment can be observed in weaker performance in rhythm perception, rhythm production and synchronization tasks. Supporting these claims, DD children’s performance in rhythm and particularly beat perception and production tasks is associated with deficits in rise time processing, phonological awareness and reading^9,11,14,36–38^. Furthermore, these associations seem to persist through adulthood^39^. Poor rhythmic skills have also been found in children with Developmental Language Disorder (DLD), who show impaired performance in speech and musical rhythm processing and tapping to a beat^16^. Additionally, more atypical groups have been found to have difficulties with rhythmic tasks: children with ADHD^40,41^, and stuttering^42,43^ leading some authors to hypothesize a common rhythmic deficit as a risk factor in these pathologies^44,45^.

A common task used to measure individuals’ sensorimotor synchronization (SMS) performance is finger tapping to auditory stimuli, in which participants need to synchronize their taps to the beat of a metronome or complex music. Although adults synchronize their movements to the sound of a beat with ease, and sometimes spontaneously, unaware of doing so^46,47^, this ability does not emerge in the first years of life and consolidates even later in middle childhood. Newborns as old as 1–2 days have been shown to process changes in musical rhythm and tempo using electrophysiological methods^48,49^. Infants already have the predisposition to move to the sound of music and can produce repetitive, isochronic movemePAnts, but they are unable to synchronize to an external beat^50^, although they have been shown to react to the tempo of music by some adjustment of their movements^51^. True synchronization behaviour has been confirmed as early as 3.5 years^52,53^ from which age children’s tapping variability and asynchrony (the difference between their taps and the reference) gradually decrease together with their preferred or spontaneous motor tempo (SMT). Children under 6 years tend to be more successful near their SMT, around 150–200 beats per minute (bpm). Around the age of 6–8, SMT decreases to around 120 bpm, approaching the average adult tempo of ca. 100 bpm^54,55^. They are also more successful in synchronizing their taps to tempi further away from their SMT and executing their tapping more consistently (with lower variability compared to their own tapping) and in synchrony with the beat, presumably reflecting the maturation of the nervous system^55–57^. In a recent study^58^ 305 6–11-year-old TD children were investigated using several tapping tasks in different tempi. Although a cross-sectional inquiry, their results shed some light on developmental changes of tapping asynchrony and variability. Developmental effects were reported for tapping variability but not for asynchrony between ages 6 and 11 regardless of stimulus tempo.

Tapping tasks have not only been shown to relate to phonological skills, reading and spelling performance in a number of studies^1,6,27,59^ but were also successful in predicting children’s future literacy skills. Norwegian first graders’ tapping was found to predict their end-of-the-year reading levels^60^, while SMS and rhythm reproduction were related to Italian 8–11-year-old’s reading, phonological awareness and rise time perception^9^. In an earlier study^61^ we found that 6–7-year-old first graders’ SMS performance predicted their literacy skills at the beginning of their second school year controlling for general cognitive abilities and RAN. Musical trials were associated with reading accuracy, phonological awareness, spelling and metronome trials with reading fluency. Testing children in their first school-year seems a promising approach for identifying poor readers prior to reading acquisition as a potential screening method.

In our present study as a follow-up to our earlier inquiry, we wanted to investigate (Q1) how children’s tapping performance changed from first to third grade in the time window (ca. 6–9 year olds) where developmental effects have already been shown^54,55,58^, and (Q2) whether children’s first-year tapping can predict third-year reading, spelling and phonological awareness scores, as children’s third-grade tapping has been shown to remain a significant predictor of their literacy in a recent cross-sectional study, although not through the mediation of phonological awareness (PA)^27^.

Based on previous studies we expected to find developmental changes in children’s tapping measures resulting in higher consistency and lower asynchrony in 3rd grade. We also hypothesized that children’s first-year tapping performance would account for some variance in their third-year literacy skills in addition to general cognitive abilities.

## Methods

### Participants

Thirty-seven third graders (mean age = 8.9; SD = 0.4; 18 girls) were recruited as participants of our earlier study^61^. All of them were typical developing first-grade children from a Budapest primary school, who spoke Hungarian as their first language. None of them reported having any learning disabilities or neurological or perceptual disorders. Studies were conducted in accordance with the Declaration of Helsinki. The study was approved by the Research Ethics Committee of Eötvös Loránd University Faculty of Education and Psychology (ref. number 2020/356), all methods were performed in accordance with the relevant guidelines and regulations. Participants were only included in the study after their parents or legal guardians gave their informed written consent.

### Equipment

For the tapping task, we used an AKAI LPD8 MIDI controller for recording participants’ tapping, and a Steinberg U-22 sound card with Audio-Technica ATH-T200 closed headphones for stimulus presentation. Steinberg Cubase 5 software was used to process tapping data.

### Measurements

#### Tapping tasks

The tapping task consisted of the presentation of an isochronous auditory sequence (metronome ticks) of three different tempo trials (80, 120 and 150 bpm). A woodblock sound from the Cubase5 database was used as a metronome tick. The different tempo trials were presented in pseudo-random order. Participants were asked to tap along with the stimulus for 30 s and continue tapping for another 30 s after the stimulus ended (synchronization-continuation design). Children were instructed in detail about the requirements of the task and were given a short demonstration. They were only given the first trial once they understood what was required of them.

Temporal data were extracted from the recordings: only the time of each tap was kept. We excluded the first 10 taps from further analysis and on the remaining taps we conducted a Rayleigh’s test to see whether participants’ tapping significantly differed from normal distribution in a particular trial. Trials that showed random tapping (a total of 12 of 222, 5.4%) were removed from the final dataset. In the continuation (unpaced) phase a maximum of 30 taps were kept for the analysis and trials under 10 valid taps were excluded (a total of 1 trial). Inter-tap intervals (ITI) from all trials were calculated and analyzed. In order to clean the dataset, ITIs smaller than the first quartile minus three times the interquartile range (Q1 – 3 × IQR) or greater than the 3rd quartile plus three times the interquartile range (Q3 + 3 × IQR) were identified as outliers and were excluded (a total of 559 of 12,579, 4.44%). Three measures were calculated for all tapping trials: Synchronization Asynchrony, Synchronization Consistency and Continuation Tapping Score.

##### Synchronization asynchrony

Synchronization asynchrony (the opposite of accuracy) was calculated as the mean of the absolute values of the differences between single taps and the nearest target beats of the stimulus. To compare asynchrony in different tempi we divided the score by the stimulus tempo giving us a percentage where 0 stands for total synchrony with the beat.

##### Synchronization consistency

The synchronization consistency measure shows how consistent a participant's tapping is independent of the stimulus in the paced tapping phase of a trial. To calculate consistency we applied circular statistics, a method frequently used for analyzing cyclical data^43^. Each individual tap was represented as a point on the circumference of a unit circle where 0° represents perfect synchrony while 180° antiphase tapping. A resultant vector (R) was calculated from all taps in a single trial, where the R vector’s length showed a participant’s tapping consistency, a hypothetical value of 1 meaning total consistency and 0 total inconsistency.

##### Continuation score

For the continuation phase, the same analyses were applied using the references of the synchronization phase, however for a substantial proportion of the trials (73%) the Rayleigh test was not significant, signalling random distribution of the participant’s tapping. Because of the high ratio of invalid trials, a binary measure was calculated based on the Rayleigh test: trials showing non-random distribution were considered successful, while those with a significance level above 0.05 unsuccessful.

##### Spontaneous motor tempo

In the spontaneous motor tempo (SMT) children were asked to tap at a comfortable tempo for 30 s. The first 10 taps were discarded and a maximum of 40 were kept in the analysis. Trials that had less than 10 valid taps were excluded (5 of 74, 6.76%). The average ITI was used as the measure of participants’ preferred tempo. Similar to the paced tapping tasks ITIs were gathered and those smaller than the first quartile minus three times the interquartile range (Q1 − 3 × IQR) or greater than the 3rd quartile plus three times the interquartile range (Q3 + 3 × IQR) were identified as outliers and were excluded (46 of 3908, 1.18%).

Tapping measures (Tapping Consistency, Tapping Asynchrony, Continuation tapping score and Spontaneous Motor Tempo) are given in raw scores to allow for better investigation of tapping performance’s change over time.

#### General cognitive abilities

Participants completed three subtests from the WISC-IV intelligence scale^62,63^. In the Block Design subtest, which measured their nonverbal reasoning abilities children were asked to recreate patterns seen on coloured sheets using bicolour wooden cubes within a time limit. As a measure of their working memory, participants were instructed to repeat progressively lengthening series of numbers first in identical and afterwards in reverse order in the Digit Span subtest. The sum of the two tasks was used as a composite score. Standard scores were calculated for each subtest using the WISC-IV manual.

#### Reading and language tests

Children’s reading performance was assessed using four tasks from Dyslexia Differential Diagnosis, Maastricht, Hungarian adaptation (3DM-H)^64,65^: Reading, Spelling, Phoneme Deletion, Rapid Automatized Naming. The Reading task consisted of reading for 30 s high-and low-frequency words and pseudo-words with increasing word length. The Spelling task consisted of a series of incomplete words shown on the computer screen and simultaneously presented auditorily to the participants who needed to choose from 4 possibilities to complete them. In the Phoneme Deletion subtest children were presented 27 pseudowords through their headphones with increasing complexity after which they were instructed to repeat them while leaving out given phonemes. In the Rapid Automatized Naming (RAN) subtest, three series of images (letters, numbers and pictures) were shown two times to the participants, and they needed to name them as quickly as possible. We used the composite standard scores calculated by the software which combines accuracy and fluency and compares them to an age standard to give a more complex picture of the participants’ performance with a more informative measure. Finally, children were given the Vocabulary subtest from the WISC-IV in which they needed to define 36 words. Age-specific standard scores were calculated based on the quality of their definitions using the WISC-IV manual.

### Procedure

Participants were first administered the above test battery in two 45-min sessions in 2020 in their first school year and measured again in 2022, 2 years later in 3rd grade also in two sessions. Testing took part individually in a separate room for ca. 30–40 min per session. The first testing session consisted of the tapping tasks and WISC-IV subtests while in the second, children completed the 3DM-H. Although the full test battery had been administered in both first and third grade to each participant, only measures that are part of the present analyses are reported in Tables 1, 2 and 3.

**Table 1:** Descriptive statistics for measures used in the analyses in 1st grade.

Measures	M	SD
RAN	57.86	9.63
Digit span	11.06	2.14
Block design	12.03	3.48
Vocabulary	14.68	3.01
Tapping consistency (80 bpm)	0.864	0.079
Tapping consistency (120 bpm)	0.874	0.066
Tapping consistency (150 bpm)	0.728	0.212
Tapping asynchrony (80 bpm)	0.126	0.053
Tapping asynchrony (120 bpm)	0.157	0.068
Tapping asynchrony (150 bpm)	0.147	0.068
Spontaneous motor tempo	115.63	26.27

**Table 2:** Descriptive statistics for measures used in the analyses in 3rd grade.

Measures	M	SD
Reading	51.7	10.69
Phonological awareness	48.3	7.05
Spelling	47.92	10.18
Tapping consistency (80 bpm)	0.877	0.108
Tapping consistency (120 bpm)	0.859	0.106
Tapping consistency (150 bpm)	0.779	0.177
Tapping asynchrony (80 bpm)	0.167	0.124
Tapping asynchrony (120 bpm)	0.091	0.058
Tapping asynchrony (150 bpm)	0.065	0.038
Spontaneous motor tempo	114.68	38.31

**Table 3:** Contingency table of continuation tapping scores in 1st and 3rd grades.

Grade	Continuation tapping score	80 bpm		120 bpm		150 bpm	
		n	%	n	%	n	%
1st	Succesful	5	2.30%	13	5.90%	11	5%
	Unsuccesful	32	14.40%	24	10.80%	26	12%
3rd	Succesful	5	2.30%	8	3.60%	10	4.5%
	Unsuccesful	32	14.40%	29	13.10%	27	12.2%

### Data analysis

IBM SPSS, Version 23 (IBM Corp., 2015), JASP (Version 0.13.1; JASP Team, 2020), R Studio (R Core Team, 2016) and Circular Package for R^66^ were used for data analysis. We removed outliers from each variable above Q3 + 1.5 × IQR or below Q1 – 1.5 × IQR. For testing normality, we used the Shapiro–Wilk method. Two-way repeated measures ANOVAs were conducted to compare children’s 1st and 3rd-grade performance scores in paced tapping trials to three tempi (80, 120 and 150 bpm). Post hoc analyses were performed using the Bonferroni correction for multiple comparisons. Estimate effect sizes are reported using partial eta squared values (*η*_*p*_^2^). A loglinear analysis was conducted to compare children’s continuation tapping performance in 1st and 3rd grade, across the three tempi.

To test the change in children’s preferred tempo over time we used a paired sample t-test comparing their preferred tempo in the 1st and 3rd grades. Furthermore, we applied multiple linear regression modelling to explore which 1st-grade measures predicted 3rd-grade reading and language outcomes. The stepwise method was used with variables entered at the *p*-value of 0.05 and excluded at 10. To determine whether the sample size was adequate to interpret our multiple regression models a post hoc power analysis was carried out using the GPower software^67^. Both significant models showed an acceptable level of statistical power: 0.94 for the first and 0.99 for the second (see Supplementary Materials for parameters).

## Results

### The effects of stimulus tempo and grade on tapping performance

**Figure 1:**
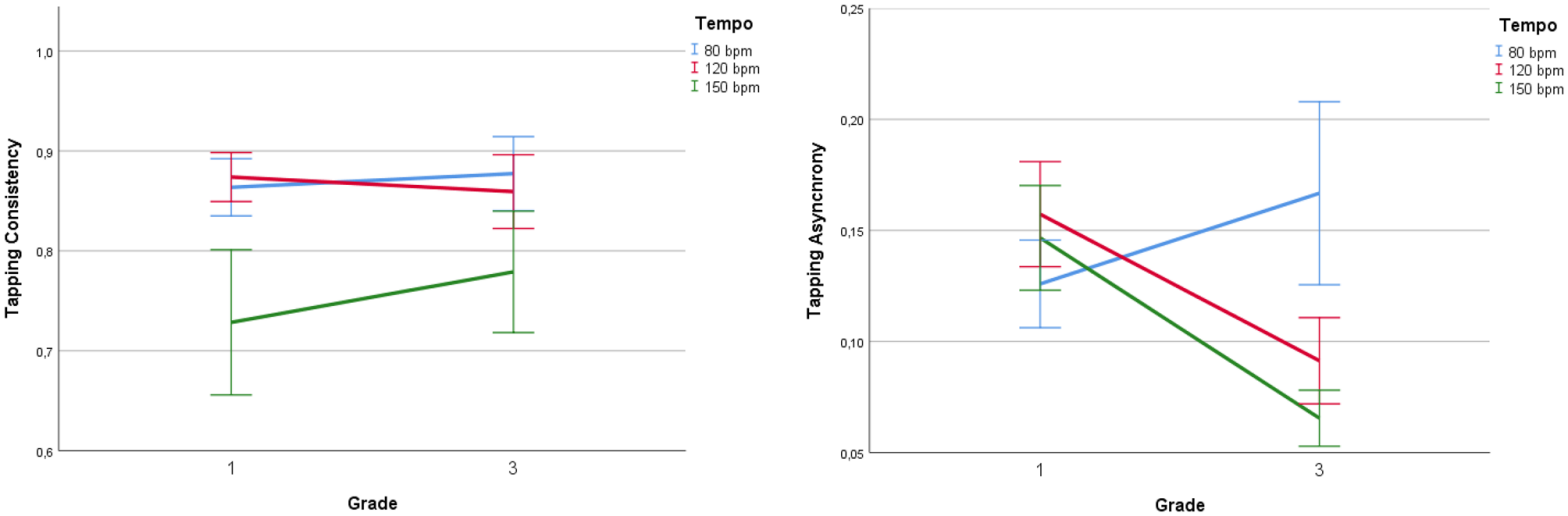
Changes in tapping measures across three tempi (80, 120, 150 bpm) from first to third grade. Error bars represent the 95% confidence interval.

Descriptive statistics for observed measures that were part of the present analyses are reported in Tables 1, 2 and 3. Two repeated measures ANOVAs were conducted for variables Tapping Consistency and Tapping Asynchrony with factors Tempo (80 bpm, 120 bpm and 150 bpm) and Grade (first and third) (see Fig. 1). For Tapping Consistency a significant effect of Tempo *F*(1, 22) = 7.482, *p* = 0.02 *η*_*p*_^2^ = 0.254 was found, with no interaction. Post-hoc comparisons revealed a difference between the three tempi: 150 bpm trials showed lower consistency compared to 120 bpm (*p* = 0.018) and to 80 bpm (*p* = 0.002) trials but no difference between 80 and 120 bpm. No significant effect of Grade was found for Tapping Consistency. We found significant effects of both Grade *F*(1, 27) = 9.110, *p* < 0.005, *η*_*p*_^2^ = 0.252 and Tempo *F*(2, 27) = 4.443, *p* < 0.016, *η*_*p*_^2^ = 0.141 for Tapping Asynchrony with a significant interaction between the two factors *F*(2, 27) = 20.243, *p* < 0.001, *η*_*p*_^2^ = 0.428. Post-hoc analysis showed that while children’s tapping asynchrony decreased over time in the 120 bpm (*p* = 0.005) and 150 bpm (*p* < 0.001) trials, it did not show a significant change for the 80 bpm trials. There was no significant difference between tempi in first grade, but in third grade, asynchrony was higher for 80 bpm compared to both 120 bpm (*p* < 0.001) and 150 bpm (*p* < 0.001) trials. A three-way loglinear analysis was conducted to examine the effects of Grade and Tempo on Continuation Tapping Scores. The analysis produced a final model that retained the Tempo × Continuation Tapping Score interaction. The likelihood of this model was *χ*^2^(6) = 1.741, *p* = 0.94. The Tempo × Continuation Score interaction was significant *χ*^2^(2) = 6.514, *p* = 0.04. This interaction indicates that the success of continuation tapping was not equal across tempi, the ratio in the 80 bpm trials was lower than in the other two (120 and 150 bpm). The chances of successful continuation tapping in either of the latter were 2.54 times higher than in the 80 bpm condition. A paired sample t-test was carried out to explore the change in children’s spontaneous motor tempo. No change in SMT showed in our analysis *t*(31) = − 0.42, *p* = 0.677; *d* = − 0.07 with children’s mean tapping rate being stable across the first and third years.

### Using 1st-grade measures to predict 3rd-grade literacy scores

**Figure 2:**
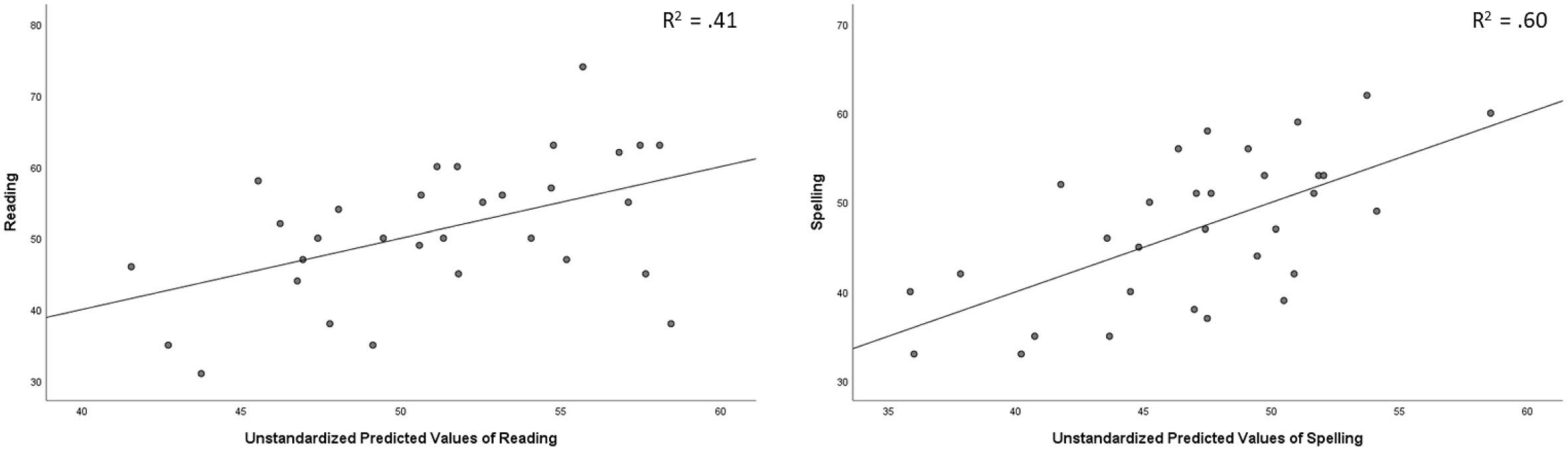
Scores and unstandardised predicted values of linear models for Reading and Spelling.

To explore which first-year measures could successfully predict children’s literacy scores in their third grade we used multiple linear regression modelling for three target measures: Reading, Spelling and Phonological Awareness (Table 3 and Fig. 2). Potential predictor variables were: Block Design, Digit Span, Vocabulary, RAN and the above-mentioned tapping measures (Tapping Consistency, Tapping Asynchrony and Continuation Tapping Score) in three different tempi (80, 120 and 150 bpm). We entered these variables using the stepwise method from which two significant models emerged. Table 4 shows the coefficients of the final models. Model diagnostics were carried out to see if the necessary assumptions were met. To control for multicollinearity, variance inflation factor (VIF) was calculated for both included and excluded variables. We found that VIF has not exceeded the value 5 commonly thought of as acceptable in any case. An additional correlation analysis was carried out to explore the relationship between the predictors (see Supplementary Material).

**Table 4:** Regression coefficients of the final linear models for reading and spelling.

Variable	Reading									Spelling				
	B			β		SE				B		β		SE
Constant	− 33.29					22.98		Constant		− 24.555				15.787
Tapping Consistency (80 bpm)	69.69**			0.50		23.23		Tapping consistency (80 bpm)		46.91**		0.39		16.565
RAN	0.44*			0.43		0.17		Digit span		2.672***		0.66		0.556
R^2^	0.41**							R^2^		0.60**				

*p < 0.05 **p < 0.01 ***p < 0.001.

A significant model was found for Reading *F*(2, 23) = 7.414, *p* = 0.004, *R*^2^ = 0.414, with Tapping Consistency (80 bpm) (*t* = 3.00, *p* = 0.007) and RAN (*t* = 2.59, *p* = 0.017) as significant predictor variables. The analysis of standard residuals showed that the data contained no outliers (Std. Residual Min = − 1.931, Std. Residual Max = 2.174) and met the assumption of independent errors (Durbin-Watson value = 2.187). The assumption of non-zero variances was met for all variables in the model (Reading Variance = 129,49; RAN Variance = 92.69; Tapping Consistency (80 bpm) = 0.006). The histogram of standardized residuals indicated a normal distribution of errors, supported by the normal probability plot of standardized residuals which showed only reasonable aberration from the line. The assumption of homogeneity of variance and linearity were also met according to the scatterplot of standardized predicted values. For Spelling a significant model emerged *F*(2, 23) = 15.979, *p* < 0.001, *R*^2^ = 0.0.60, with Digit Span (*t* = 4.808, *p* < 0.001) and Tapping Consistency (80 bpm) (*t* = 2.832, *p* = 0.01) as significant predictors.

The analysis of standard residuals showed the data to be void of outliers (Std. Residual Min = − 1.511, Std. Residual Max = 1.709). The assumption of independent errors was met according to the Durbin-Watson value (2.029). The assumption of non-zero variances was not violated (Spelling Variance = 110.86; Digit Span Variance = 4.57, Tapping Consistency (80 bpm) Variance = 0.006). Looking at the histogram of standardized residuals an approximately normal distribution of errors was observed. The P–P plot of standardized residuals indicated an acceptable deviation from the line. The scatter plot of standardized predicted values showed that the assumptions of homogeneity of variance and linearity were not violated. For Phonological Awareness, the regression analysis did not result in a significant model.

## Discussion

In our present study, we measured tapping, general cognitive abilities and language and literacy skills of 3rd-grade children who took part in a similar examination two years before. Comparing their scores we focused on two questions: How their tapping changed from first to third grade (Q1), and whether their first-grade tapping could predict their present literacy scores (Q2).

### The effect of tempo and grade in children’s tapping performance

Looking at tapping performance, a general effect of tempo could be observed in all measures (consistency, asynchrony and continuation tapping score), while a developmental effect was only found in the case of asynchrony, although not for all tempi. Improvement in asynchrony has been found in earlier studies with similar age groups^55,56,68^, but this is in contrasts with the findings of Carrer et al.^58^, who found that while tapping variability decreases with age regardless of tempo between 6 and 11 years of age, synchronization error does not change in this interval. The stability of the consistency measures over time in contrast to asynchrony can presumably be accounted for by the fact that children’s ability to tap consistently emerges earlier in their development than their ability to adapt to external stimuli^50,55–57^ thus their performance in first grade has already reached a high level that does not allow for further improvement in this developmental period.

Examining the role of stimulus tempo we found that higher tempo (150 bpm) had a deteriorating effect on tapping consistency. A feasible explanation could be the higher demand on motor execution. At the same time asynchrony did not increase with tempo conceivably because negative mean asynchrony has been shown to decrease or disappear with higher stimulus tempo^69^. However, lower tempo (80 bpm) did have an effect on tapping asynchrony. In general, we found that as asynchrony for 120 and 150 bpm trials decreased with age, it remained stable for 80 bpm resulting in weaker performance compared to the other trials in 3rd grade while they were on approximately the same level in 1st grade. Adjusting their tapping to an external tempo slower than their SMT has been reported to cause difficulty to this age group^68^. Although weaker performance in tempi distant from children’s SMT is well is a well-documented phenomenon^55,70^ we hypothesize that the task of adapting to slower tempi might put a higher load on executive functioning, in particular inhibition, as they have to resist the general tendency of speeding up.

There was no significant change in children’s spontaneous motor tempo between the two measurements. We found an average SMT of 116 bpm in contrast to 115 bpm in first grade. Our results support earlier findings of children’s preferred tempo, as both are around reported values^54,55^. Our findings also show that while there might be a developmental change in SMT around 6 years, our sample, who were ca. 7 years old at the time of the first measurement and 9 years old at the second, already showed consolidated average tempo and no significant change in the following 2 years. Concerning the relevance of these findings, it should be noted that while most of our knowledge about the developmental trajectory of tapping performance comes from cross-sectional studies our results may give a more detailed picture of this age group from a longitudinal view.

### Predicting children’s 3rd-year literacy skills

To examine if first-year tapping performance could predict third-year literacy scores, we built multiple linear regression models using tapping, general cognitive, language and literacy measures as independent variables. Children’s reading was predicted best by a model with Tapping Consistency (80 bpm) and RAN accounting for 41% of the variance. Although RAN is a well-established predictor of reading in the literature the tapping variable’s contribution to the model was slightly larger (β = 0.5 and 0.43). This result is not only consistent with earlier studies^9,60^ but also with our first-year results when children’s reading fluency was best predicted by a similar tapping consistency measure^61^.

Our final model predicting Spelling performance in third grade included Digit Span and Tapping Consistency (80 bpm) accounting for 60% of the variance in Spelling. Although not the most common predictor, working memory has been found in several cases to relate to spelling performance supposedly in tasks where serial order is included^71,72^. Our results may also support the conclusions of an earlier study with Hungarian children^73^, in which the authors hypothesized that the process of spelling includes applying learned grammatical rules to word representations which assumes a satisfactory level of working memory. Concerning the predictors of Spelling performance, it would be important to take into account possible language specific differences: for example, Hungarian language has a shallow orthography, and this might contribute to the predictors structure. Evidently, more cross-linguistic studies are needed to further clarify these differences.

No significant model could be found for predicting Phonological Awareness. Although the mediating role of PA between tapping and early literacy skills is well supported by the literature^28,32,34^, the present findings are well in line with those of Lê et al.^27^ who found that 3rd graders’ reading was directly connected to their tapping without the mediation of PA. The role of phonological skills has been shown to diminish as children’s reading develops and other predictors, for example, RAN gain more importance^74–76^.

We assume that the connection uncovered in the present inquiry might not be mediated by phonological skills, as we found earlier in first grade^61^, but by some domain-general mechanisms (e.g. executive functioning, attention or statistical learning). Inhibition would seem a good candidate as it has been proposed as a general skill behind the transfer effects of musical training^77^. The distinct role of the slow tempo tapping consistency measure could also be interpreted in this way. Children with more developed inhibition control in first grade were better able to adapt to the slower external beat by overcoming their tendency to speed up and keep a consistent motor tempo at a slower speed than their SMT, while this skill also resulted in more precise and fluent reading and better performance in the spelling task, in which they had to analyze and manipulate word representations.

Our results show that the relation between tapping performance and literacy skills, found in first grade persists throughout the third school year (and conceivably further), making the sensorimotor synchronization task a potentially effective instrument for predicting literacy outcomes, and a useful tool for early screening of reading difficulties.

## Conclusion

The results of the present study show that children’s tapping performance measured in their first school year can significantly predict their third-grade reading and spelling skills. Slow tempo (80 bpm) Tapping consistency, which we found to not show a developmental effect in this period, was a successful predictor, while asynchrony measures, generally showing significant improvement, did not contribute to our models. Future research should focus on clarifying the relationship and possible mediating effects between early rhythmic and linguistic predictors of literacy in a longitudinal design to help identify at-risk children and inform the development of future rhythm-focused training protocols. Further investigations in longitudinal design should be conducted to give support to the present findings with a wider variety of cognitive measures including tests of executive functioning.

## Supplementary Information

Supplementary Tables.

## Data Availability

The datasets generated during and/or analysed during the current study are available from the corresponding author on reasonable request.
