# Supplementary material for: First school year tapping predicts children's third-grade literacy skills
Source: Sci Rep. 2023 Feb 9;13:2298. doi: 10.1038/s41598-023-29367-5 (PMC9911382; doi:10.1038/s41598-023-29367-5)
Supplement: Supplementary file 1 — Supplementary Tables. [file 41598_2023_29367_MOESM1_ESM.docx]

**Supplementary table 1.**
Parameters of post hoc power analysis were carried out on the two regression models.

|  | Sample size | Effect size (f^2^) | Power (1-β prob) |
| --- | --- | --- | --- |
| Model 1 | 24 | 0.71 | .94 |
| Model 2 | 24 | 1.52 | .99 |

Note. α = .05. The number of predictors for both models is 2.

|  | Digit Span | | Block Design | | Vocabulary | | RAN | | Consistency  (80 bpm) | | Consistency (120 bpm) | | Consistency  (150 bpm) | | Asynchrony (80 bpm) | | Asynchrony (120 bpm) | | Asynchrony (150 bpm) | | |
| --- | --- | --- | --- | --- | --- | --- | --- | --- | --- | --- | --- | --- | --- | --- | --- | --- | --- | --- | --- | --- | --- |
| Digit Span | — |  |  |  |  |  |  |  |  |  |  |  |  |  |  |  |  |  |  |  | |
| Block Design | 0.173 |  | — |  |  |  |  |  |  |  |  |  |  |  |  |  |  |  |  |  | |
| Vocabulary | 0.210 |  | 0.082 |  | — |  |  |  |  |  |  |  |  |  |  |  |  |  |  |  | |
| RAN | 0.174 |  | -0.045 |  | 0.306 | * | — |  |  |  |  |  |  |  |  |  |  |  |  |  | |
| Consistency (80 bpm) | 0.198 |  | -0.094 |  | 0.015 |  | -0.028 |  | — |  |  |  |  |  |  |  |  |  |  |  | |
| Consistency (120 bpm) | 0.084 |  | 0.039 |  | -0.181 |  | -0.093 |  | 0.337 | * | — |  |  |  |  |  |  |  |  |  | |
| Consistency (150 bpm) | 0.305 | * | 0.085 |  | -0.019 |  | 0.078 |  | 0.231 |  | 0.020 |  | — |  |  |  |  |  |  |  | |
| Asynchrony (80 bpm) | -0.063 |  | 0.119 |  | 0.041 |  | 0.175 |  | -0.457 | *** | -0.460 | ** | -0.017 |  | — |  |  |  |  |  | |
| Asynchrony (120 bpm) | -0.025 |  | -0.007 |  | 0.086 |  | 0.239 |  | -0.118 |  | -0.338 | ** | 0.050 |  | 0.305 | * | — |  |  |  | |
| Asynchrony (150 bpm) | -0.226 |  | -0.020 |  | 0.056 |  | -0.013 |  | -0.164 |  | -0.233 |  | -0.348 | ** | 0.166 |  | 0.270 | * | — |  | |
| * p < .05, ** p < .01, *** p < .001  Note. Kendall’s Tau-b values are reported. | | | | | | | | | | | | | | | | | | | | |  |

**Supplementary table 2.**
Correlations between predictor variables entered in the linear models.
